# A synthetic peptide rescues rat cortical neurons from anesthetic-induced cell death, perturbation of growth and synaptic assembly

**DOI:** 10.1038/s41598-021-84168-y

**Published:** 2021-02-25

**Authors:** Fahad Iqbal, Marcus Pehar, Andrew J. Thompson, Urva Azeem, Kiana Jahanbakhsh, Nerea Jimenez-Tellez, Rasha Sabouny, Shadab Batool, Atika Syeda, Jennifer Chow, Pranav Machiraju, Timothy Shutt, Kamran Yusuf, Jane Shearer, Tiffany Rice, Naweed I. Syed

**Affiliations:** 1 University of Calgary, Calgary, T2N 4N1 Canada; 2 University of Oxford, Oxford, UK

**Keywords:** Cell biology, Neuroscience, Physiology, Medical research, Paediatric research, Preclinical research, Translational research

## Abstract

Anesthetics are deemed necessary for all major surgical procedures. However, they have also been found to exert neurotoxic effects when tested on various experimental models, but the underlying mechanisms remain unknown. Earlier studies have implicated mitochondrial fragmentation as a potential target of anesthetic-induced toxicity, although clinical strategies to protect their structure and function remain sparse. Here, we sought to determine if preserving mitochondrial networks with a non-toxic, short-life synthetic peptide—P110, would protect cortical neurons against both inhalational and intravenous anesthetic-induced neurotoxicity. This study provides the first direct and comparative account of three key anesthetics (desflurane, propofol, and ketamine) when used under identical conditions, and demonstrates their impact on neonatal, rat cortical neuronal viability, neurite outgrowth and synaptic assembly. Furthermore, we discovered that inhibiting Fis1-mediated mitochondrial fission reverses anesthetic-induced aberrations in an agent-specific manner. This study underscores the importance of designing mitigation strategies invoking mitochondria-mediated protection from anesthetic-induced toxicity in both animals and humans.

## Introduction

Anesthetics are required for major clinical procedures as they enable complex surgeries with high levels of patient safety. Recent studies—including important work by Jevtovic-Todorovic, et al.^1^—have shown that several clinically used anesthetics also exert significant neurotoxic effects on both animal models^1–13^ and human neural stem cells^14,15^. The evidence of harm ranges from cell death^3,15–18^ to suppression of neurite outgrowth^19,20^ to perturbation of synaptic connectivity^7,10–12^. Moreover, some epidemiological studies have also implicated a variety of anesthetic agents in learning, memory and cognitive dysfunction in children, although the clinical evidence remains equivocal^21–26^.

Our understanding of the mechanisms of how various anesthetics function has vastly improved in recent years^21,27,28^, but a complete picture of their modes of action in the context of the developing brain remains blurry—owing mainly to the complexity of the brain and the ethical implications of using humans as experimental subjects. On the other hand, in vitro animal models are much less complex, with rodent brains also having far shorter developmental periods. Using these robust models, anesthetics have been shown to impact social behavior in mice^29^, cause significant learning deficits, cell death, and degeneration in rat brains^1,30^. The exposure of infant rhesus monkeys under one week of age, to isoflurane, nitrous oxide, propofol, ketamine and other anesthetics caused myriad toxic effects—ranging from cell death to cognitive deficits^31–34^. These results have since been supported by several in vitro studies which provide further mechanistic insights into the anesthetic-induced effects; these could not otherwise have been deduced in intact animals. In these models, anesthetics have been shown to alter network development, neuronal communications and morphology to varying scales, and at various levels of anesthetic concentrations and exposure times^20,35,36^. For example, low levels of propofol have been reported to cause temporary neurite retraction in cultured cortical cells^20^, while isoflurane, sevoflurane, and propofol were found to upregulate the mechanistic target of rapamycin (mTOR), suggesting common pathways underlying their impacts on developing neural networks^36^. Desflurane and sevoflurane on the other hand, when used within clinical concentration ranges have also been shown to reduce neurite growth, synaptic puncta density, and impact spontaneous synaptic currents in cultured neurons^35^. Although cell culture studies are a physiological compromise, such experiments do nevertheless serve as powerful tools which help to tease apart potential pathways while identifying sites that could then potentially be targeted for mitigation strategies.

Notwithstanding the lack of conclusive evidence of harm on human learning, memory and cognition deficits, questions over the clinical significance of anesthetic-induced neurotoxicity have nevertheless compelled both the Food and Drug Administration and Health Canada to issue warnings regarding anesthetic use for young children and pregnant mothers^37,38^. Several reviews, retrospective and patient based studies have also raised questions regarding anesthetic-induced side-effects in the clinical domain^21–26^. In the absence of definitive answers regarding the potential sites of anesthetic action and the nature of their harmful effects, strategies to mitigate their potential long-term side effects cannot, however, be put in place. Moreover, clinicians are left with conflicting data from both human subjects and animal studies due to inconsistencies in the used experimental design. Thus, a more relevant approach towards identifying key cellular targets for potential anesthetic toxicity is warranted, first in animal models in order to reconcile the discrepancies found in the published accounts, before proceeding to human studies. Of note, a direct comparison between γ-aminobutyric acid (GABA)-ergic and N-Methyl-D-aspartic acid (NMDA)-ergic general anesthetics (the major targets for most anesthetics), and the extent to which they compromise neuronal development is yet to be performed.

To provide a comprehensive and comparative account of the actions of various anesthetic agents, we examined one inhalational anesthetic, desflurane, and two intravenous compounds, propofol and ketamine. Propofol—the most commonly used intravenous anesthetic—and desflurane are known to act primarily by promoting inhibitory GABAergic synaptic transmission^21^, while ketamine acts primarily by blocking NMDA receptor-mediated excitatory synaptic transmission^21^. We examined the above agents under identical and clinically relevant conditions for their differential effects on cell viability, neurite outgrowth, and synapse formation in rat cortical neurons in order to identify potential sites for their harmful effects.

Recent studies have implicated mitochondrial dysfunction, specifically the fragmentation of its networks as a common denominator in several degenerative diseases^39–42^, and most recently in anesthetic-induced neurotoxicity^15,18,35,43,44^. We therefore sought to determine whether these agents, when used under identical and relevant conditions, affect mitochondrial networks, and if these potentially detrimental effects could be prevented by using a synthetic peptide P110, which selectively impairs dynamin-1-related protein (Drp1)-mediated mitochondrial fission^45^. Previous studies employing a general mitochondrial fission inhibitor, mdivi-1, found it to ameliorate anesthetic neurotoxicity in primary neurons^35^ and neural stem cells^15^. However, the off-target effects of this drug ranged from inhibiting cellular proliferation to respiratory impairment, and the inhibition of basal levels of fission which undermined its potential therapeutic utility^25,46,47^. Here we asked the question if P110 peptide would rescue neurons from both intravenous and inhalation anesthetic-induced toxicity.

## Methods

### Ethical approval

All animal procedures were carried out in accordance with the policies and procedures established by the University of Calgary Animal Care and Use Policy under the Canadian Council on Animal Care. All experimental protocols have been approved by the University of Calgary Animal Care Committee. All data were obtained without unnecessary pain and suffering of animals in accordance with the current best practices and used most appropriate species for the study^48^. The protocols used are largely identical to those employed in our previous study^35^ and were adapted accordingly. All in vivo work was carried out in compliance to ARRIVE guidelines.

Both male and female postnatal day 0 wild-type Sprague Dawley strain code 400 rats from Charles River Laboratories were used. The mothers of these postnatal day 0 rats were kept in a conventional room set at 21 °C on a 12-h light/dark cycle from 7:00 am to 7:00 pm local time and were fed ad libitum. Sedation was achieved via ice induced hypothermia, where the neonatal rat was wrapped in tissue paper and placed in a chamber filled with ice for 10 minutes^49^. Decapitation was performed following the loss of movement, prior to the regaining of consciousness. Following decapitation, cortical tissue was collected.

### Primary rat neuronal cell culture and P110 treatment

Sprague–Dawley rat frontal cortices were isolated from postnatal day 0 pups and enzymatically treated with papain (50 u/mL; Worthington Biochemical Corp., Lakewood, NJ, USA). The cells were then triturated in glass pipettes of decreasing size to create a uniform cell suspension and plated at appropriate confluence onto cover slips coated with laminin (2 μg/mL; MilliporeSigma, St. Louis, MO, USA) and poly-D-lysine (30 μg/mL; MilliporeSigma, St. Louis, MO, USA) in culture dishes. We aimed for a consistent monolayer of cells averaging around 9,000 to 12,000 cells/cm^2^ for “lower density” cultures depending on the experiment design^50^. Cells settled for one hour at 37 °C and 5% CO_2_ prior to the addition of additional 2 mL culture media. The culture medium used was Neurobasal medium supplemented with 2% B27 (Thermo Fisher Scientific, Waltham, MA, USA), L-Glutamine (200 mM; Thermo Fisher Scientific, Waltham, MA, USA), 4% FBS (Thermo Fisher Scientific, Waltham, MA, USA), and penicillin–streptomycin (10 000u/mL penicillin, 10 000μ/mL streptomycin; Invitrogen, Waltham, MA, USA), which was changed (50% removed and replaced) every 3 days. Some culture dishes were treated with P110 (provided by Daria Mochly-Rosen, Department of Chemical and Systems Biology, Stanford University School of Medicine, Stanford, CA, USA) for one hour in culture media, while controls had normal culture media which did not contain the peptide.

The cells were incubated at 37 °C and 5% CO_2_ for 1 h before anesthetic exposure/treatment.

### Desflurane exposure

After 1-h incubation at 37 °C and 5% CO_2_, the cells were exposed to 0.5 minimum alveolar concentration (MAC) equivalent of desflurane (4.3 Vol%; Baxter Corporation, Mississauga, ON, Canada) in an airtight modular incubator chamber (Billups-Rothenberg) for one hour to mimic the inhalation. Desflurane-medical air gas mixtures were vaporized using a Datex-Ohmeda Aestiva/5 vaporizer and concentrations were monitored with a GE Healthcare Gas Analyzer. Controls were exposed to medical air only (79% Nitrogen, and 21% Oxygen; Air Liquide). After 1 h of desflurane-medical air mixture or just medical air exposure, the neurons were placed back and maintained in an incubator (37 °C, 5% CO_2_) until use. This exposure time was consistent with previous studies, aimed at optimal gas exchange with minimum out of incubator time for culture neurons.

### Propofol and ketamine treatment

After 1-h incubation at 37 °C and 5% CO_2_, propofol and ketamine were added to the experimental treatments individually at appropriate concentrations (10 µM propofol and 5 µM ketamine, respectively). The choice of these concentrations was based on preliminary findings and previous studies wherein they were deemed to be the lowest to have an effect on neuronal structural and functional characteristics. These corresponded with clinically used concentrations when administered intravenously to humans^13,14,18^. As a range of 1-10mcg/mL of blood propofol concentrations is expected in humans under anesthesia, converting this to µM gives a range between 5.6 µM and 56µM^51^. Accordingly, this led to the choice of 10 µM throughout our propofol experiments. For ketamine, the blood concentration for anesthesia ranges between 2000 and 3000 ng/mL, which equates to 7.3–10.9 µM. A dose response conducted during our preliminary investigation suggested doses ranging from 5 to 10 µM initiated varying degrees of cytotoxic effects. Following initial anesthetic treatment, the drugs were then further diluted with half media replacement the next day. The cells were maintained in an incubator (37 °C, 5% CO_2_) until use.

### Live-cell fluorescent imaging and confocal microscopy

To assess the impact of desflurane on the morphological integrity of the mitochondria, cells were grown for 4 days post-culture and then had their media replaced with media containing MitoTracker Red CMXRos (ThermoFisher Scientific, cat. M7512) (200 nM) and incubated with the cells for 20 min at 37 °C and 5% CO_2_. The cells were then washed 3 times with DPBS, and fresh media was added. The cells were then imaged.

To simultaneously assess the impact of anesthetics on mitochondrial morphology and superoxide (SO) production, cells were grown for 4 days post-culture and then had their media replaced with that containing MitoTracker Green (ThermoFisher Scientific, cat. M7514) (70 nM) and MitoSOX Red (ThermoFisher Scientific, cat. M36008) (5 µM) and incubated with the cells for 20 min at 37 °C and 5% CO_2_. The cells were then washed 3 times with DPBS, and fresh media was added. The cells were then imaged.

**Figure 1:**
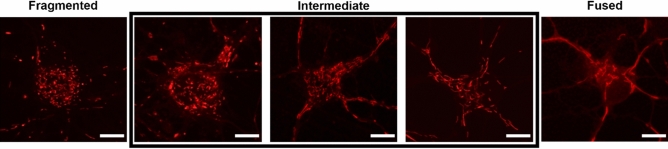
Mitochondrial morphology quantification reference scale. Cells were manually classified into one of 3 categories. Cells which exhibited more extreme fragmentation or hyper-fusion were counted to their closest equivalent. Neurons labeled with Mitotracker Red CMXRos. Scale bars indicate 10 µm.

Neurons were imaged at this time point to minimize glial interference that would complicate neuronal identification. Mitochondrial morphology was quantified by assessing the percentage of cells that exhibited a particular morphology throughout at least three biological replicates. The individual acquiring images and data analysis was blinded to the experimental condition in a randomized manner. Only cells with very clear and distinguishable mitochondrial network morphologies were selected. Image analysis was also performed double-blinded. Each cell was classified as having a given mitochondrial morphology based on the predominant mitochondrial morphology present in each cell according to the following criteria^35^: mitochondria were considered fragmented when less than 0.75 μm in length, intermediate when 0.75 μm to 3 μm in length, and fused when greater than 3 μm in length. These characterizations were reflected on a three-point fragmentation scale and cells were classified as having fused, intermediate, or fragmented mitochondrial networks (Fig. 1).

Fluorescence images were taken with an Olympus SD-OSR spinning-disk confocal microscope with a mounted incubation system (Olympus Corp.) using the same imaging parameters for every dish. A 100 × objective was used. Mitochondrial SO production was measured by calculating the mean grey intensity of SO signal per field of view and compared between treatments using the same imaging parameters and thresholds in ImageJ. The intensity of SO signal that localized to the MitoTracker Green signal was also calculated and compared in the same manner.

### Cell viability assay

The effects of anesthetics on neuronal viability were tested 3 days post-culture via the LIVE/DEAD Viability⁄Cytotoxicity Kit (Molecular Probes). This time point was selected to minimize any glial proliferation that could mask changes in viability of developing neurons. Specifically, the cells were exposed to calcein-AM (green, live cells) and ethidium homodimer-1 (red, dead cells) dyes at room temperature for 15 min and imaged using a Zeiss Axio Observer Z1 microscope (Zeiss Corp.) with a 10 × objective. The percentage of alive cells was semi-automatically counted using the Cell Counter plugin in ImageJ with the same thresholds for all the treatments and corrected for differences in signal intensity. All data collection was blinded and conducted in a standardized and systematic manner. That is, areas within a standard grid were imaged with 895 µm spacing in the x-axis and 630 µm in the y-axis. This approach was meant to prevent any selection bias while choosing a random area for imaging.

### Immunofluorescent staining

Immunofluorescent staining to assess the impact of anesthetics on synaptic network assembly was conducted on neurons grown for 10 days post-culture. Fixation was performed with 4% paraformaldehyde for 20 min at room temperature. The fixed cells were permeabilized for 1 h at room temperature with incubation media consisting of 0.1% Triton X with 5% goat serum. A primary rabbit monoclonal antibody for synaptophysin (1:500) (Abcam; EP1098Y) and mouse monoclonal antibody for PSD-95 (1:1000) (NeuroMab; 75-028) were applied to the cultures overnight at 4 °C. Secondary antibodies of AlexaFluor 561 goat anti-rabbit IgG (1:400) (Invitrogen; A11011), and AlexaFluor 488 goat anti-mouse IgG (1:400) (Invitrogen; A28175) were then applied. Dishes were mounted with MOWIOL mounting media with DAPI to stain nuclei (Sigma-Aldrich). Cells were imaged with a Zeiss Axio Observer Z1 microscope (Zeiss Corp.) with a 63 × objective over randomized areas across the cover slip. All image acquisition and data analysis experiments were double blinded where the observer was unbeknown to the nature of the experimental paradigm. Synaptic puncta density was quantified manually with a focus on primary neurites which emanated either from the cell body or secondary projections from other dendritic neurites under identical thresholds. Multiple processes from a minimum of 5 cells per biological replicate were quantified.

To assess the impact of anesthetics on neuronal cytoskeletal growth, the neurons were stained with primary chicken polyclonal antibody against the 160 kDa fragment of neurofilament (1:500) (Novus Biologicals; NB300-222) and the secondary antibody of AlexaFluor 488 goat anti-chicken IgY (1:400) (Invitrogen; A32931). The neurons were imaged using a Zeiss Axio Observer Z1 microscope (Zeiss Corp.) with a 20 × objective over a standard grid, with 435 µm spacing in the x-axis and 430 µm in the y-axis. This was meant to prevent any selection bias while randomly selecting an area for imaging. Image acquisition parameters (laser intensity, pinhole sizes, exposure times, gain settings etc.) were kept consistent throughout all treatments. Average neurite outgrowth of only those neurons present in the center of the field of view was quantified using the ImageJ plugin NeuronJ (minimum n = 19 areas of 0.4 mm^2^ for each treatment) with identical conditions and thresholds for each condition.

Antibody specificity data has been previously published via negative control experiments that show no immunofluorescence in the absence of primary antibodies^35,52^.

### Statistical analysis

Statistical significance tests were conducted with GraphPad Prism 8. One-way ANOVA was used to compare groups with one independent variable (i.e. control vs. propofol vs. ketamine). Two-way ANOVA was used to compare groups with two independent variables (i.e. P110 treatment and anesthetic treatment or exposure). Tukey's or Sidak’s multiple comparisons tests were conducted as post-hoc tests as appropriate. Values were graphed as mean ± standard deviation (SD) across at least 3 biological replicates. Differences between data were considered significant if appropriate post-hoc statistical tests resulted in *p* < 0.05. Significance parameters were indicated with asterisks as follows: * *p* < 0.05, ** *p* < 0.01, *** *p* < 0.001, **** *p* < 0.0001.

## Results

### Desflurane and propofol cause significant cell death in primary rat cortical neurons

To directly assess the effects of both GABAergic and NMDAergic anesthetics on neuronal health, and to resolve discrepancies emanating from conflicting results in the literature, we tested the effects of desflurane (4.3 vol%), propofol (10 µM), and ketamine (5 µM) on cortical neuronal viability under identical experimental conditions. This clinically relevant concentration for desflurane equates to 0.5 minimum alveolar concentration (MAC). The concentrations for the comparative agents propofol and ketamine were chosen based on their clinical utility and as per the previously published studies in various animal models^13,14,18^.

**Figure 2:**
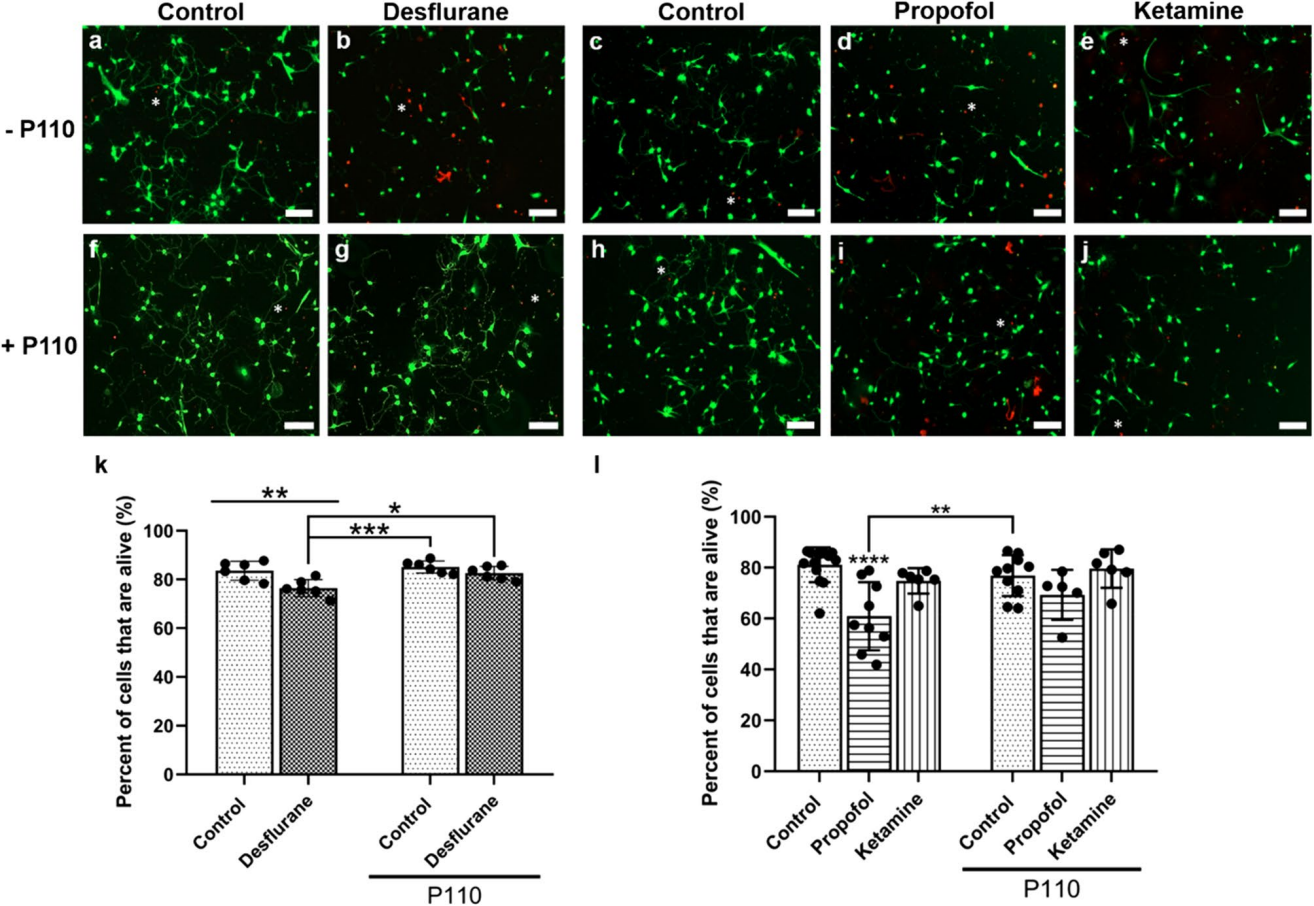
Anesthetics induced cell death in primary cortical neuronal cultures and the synthetic peptide P110 rescued neurons from anesthetic-induced toxicity. Representative live-fluorescent images of different conditions labeled with a LIVE/DEAD Viability/Cytotoxicity Assay where live cells are identified with calcein-AM (green) and dead cells are identified with ethidium homodimer-1 (red) **(a–j)**. Quantification of percentage of live cells **(k–l)**. Exposure to 4.3 Vol% desflurane, treatment with 10 µM propofol, but not treatment with 5 µM ketamine caused significant cell death. P110 alone did not affect cellular viability whereas its pre-treatment protected neurons against desflurane. P110 pre-treatment followed by propofol or ketamine did not differ from that of the control. Two-Way ANOVA. * *p* < 0.05, ** *p* < 0.01, *** *p* < 0.001, **** *p* < 0.0001 as determined by pairwise comparison of post-hoc tests. Bars indicate ± SD measured across biological replicates. Each data-point indicates the mean viability reported in any given biological replicate. Scale bars indicate 100 μm.

Cells cultured under control conditions displayed healthy, branching neurites with there being no signs of vacuolation. On the other hand, neurons exposed to desflurane, propofol, and ketamine exhibited signs of vacuolation. A quantitative analysis of these observations was conducted via live/dead assay and multiple areas (0.58 mm^2^) of three replicates of each condition were imaged and quantified under identical thresholds (Fig. 2a–j). Examples of dead cells are indicated with asterisks (Fig. 2a–e). Two-way ANOVA tests confirmed that desflurane-exposed cells exhibited significantly reduced cell viability: (*Mean* = 76.49%, *SD* = 3.49% alive, *p* = 0.006, n = 6, *d* = 1.9295) as compared to control (*M* = 83.6%, *SD* = 3.87% alive, n = 6) (Fig. 2k). Similar observations were made for the intravenous agents, which showed that propofol-treated cells exhibited increased cell death (*M* = 60.92%, *SD* = 13.35% alive, *p* < 0.0001, n = 9, *d* = 1.9063) as compared to control (*M* = 81.14%, *SD* = 6.84% alive, n = 14) (Fig. 2l). Ketamine, however, did not significantly affect cell viability compared to control (M = 74.76%, SD = 5.01% alive, *p* = 0.6774, n = 6, *d* = 1.0642) (Fig. 2l). Taken together, these data demonstrate that the anesthetics desflurane and propofol cause significant death in rat cortical rat neurons.

### Anesthetics differentially affect neurite outgrowth and synaptic network assembly in developing rat cortical neurons

#### Neurite outgrowth

**Figure 3:**
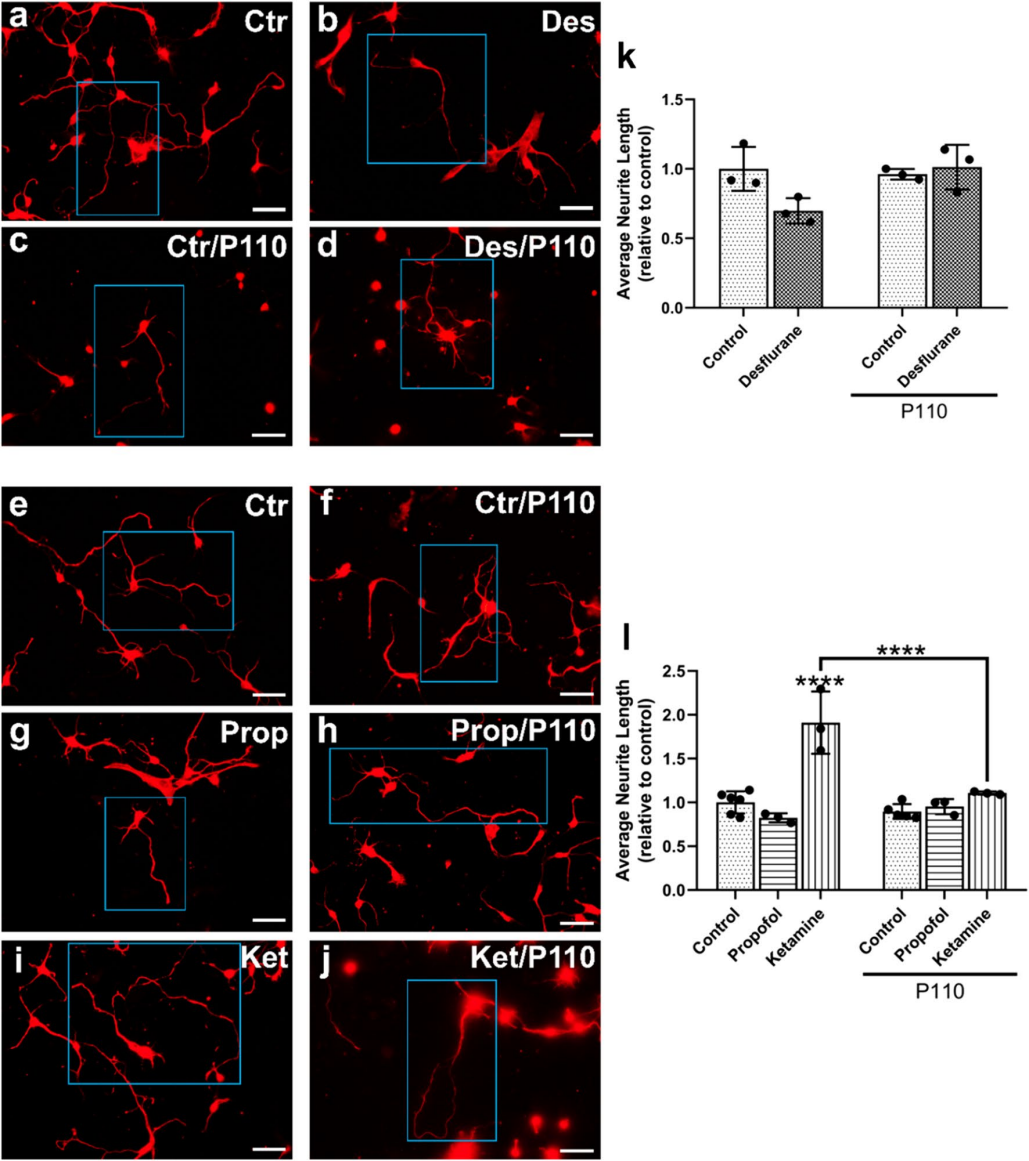
Ketamine increased average neurite outgrowth, which was attenuated by P110, whereas neither desflurane nor propofol had any significant impact. Representative fluorescent images of cortical neurons stained for neurofilament at day in vitro 3 **(a–j)**. Quantification of average neurite length relative to control **(k–l)**. Exposure to 4.3 Vol% desflurane did not decrease average neurite lengths, whereas treatment with 10 µM propofol did not significantly affect neurite outgrowth. Treatment with 5 µM ketamine resulted in enhanced growth compared to control. P110 alone did not affect neurite outgrowth wheras P110 pre-treatment mitigated ketamine-induced changes. P110 treatment followed by propofol or desflurane did not differ from control. Two-Way ANOVA. **** *p* < 0.0001 as determined by pairwise comparison of post-hoc tests. Bars indicate ± SD measured across biological replicates. Each data point indicates the mean average neurite lengths per biological replicate. Scale bars indicate 50 μm.

Developing neurons extend their axonal and dendritic processes and branch out to reach their synaptic targets; failure to do so results in perturbed connectivity and compromised functional assembly of the brain. To assess whether general anesthetics with different modes of action affect neurite growth of surviving neurons, we next tested the effects of desflurane, propofol, and ketamine on the ability of developing neurons to exhibit outgrowth. For this, the cultured neurons were stained with polyclonal antibodies against the 160 kDa fragment of neurofilament (Fig. 3a–j) and average neurite lengths were quantified in multiple areas of 0.15 mm^2^. We found that neurons exposed to 4.3 vol% desflurane exhibited neurite lengths that were not significantly lower than control (*M* = 0.70, *SD* = 0.09 fraction of control, *p* = 0.0663, n = 3, *d* = 2.3111) compared to control (*M* = 1.00, *SD* = 0.16, n = 3) (Fig. 3k). Neurons treated with 10 µM propofol also showed no significant change in neurite outgrowth (*M* = 0.82, *SD* = 0.05 fraction of control, *p* = 0.8415, n = 3, *d* = 1.8276) compared to control (*M* = 1.91, *SD* = 0.36 fraction of control, *p* = 0.0003, n = 3) **(**Fig. 3l). Neurons treated with ketamine however, exhibited more robust growth than their control counterparts (*M* = 1.91, *SD* = 0.36 fraction of control, *p* < 0.0001, n = 3, *d* = 3.3623) and compared to those treated with propofol alone (*p* < 0.0001) (Fig. 3l). Taken together, these data demonstrate that neither desflurane nor propofol inhibit average neurite outgrowth length per cell, whereas ketamine enhances neuronal outgrowth of cortical neurons.

#### Synaptic network assembly

**Figure 4:**
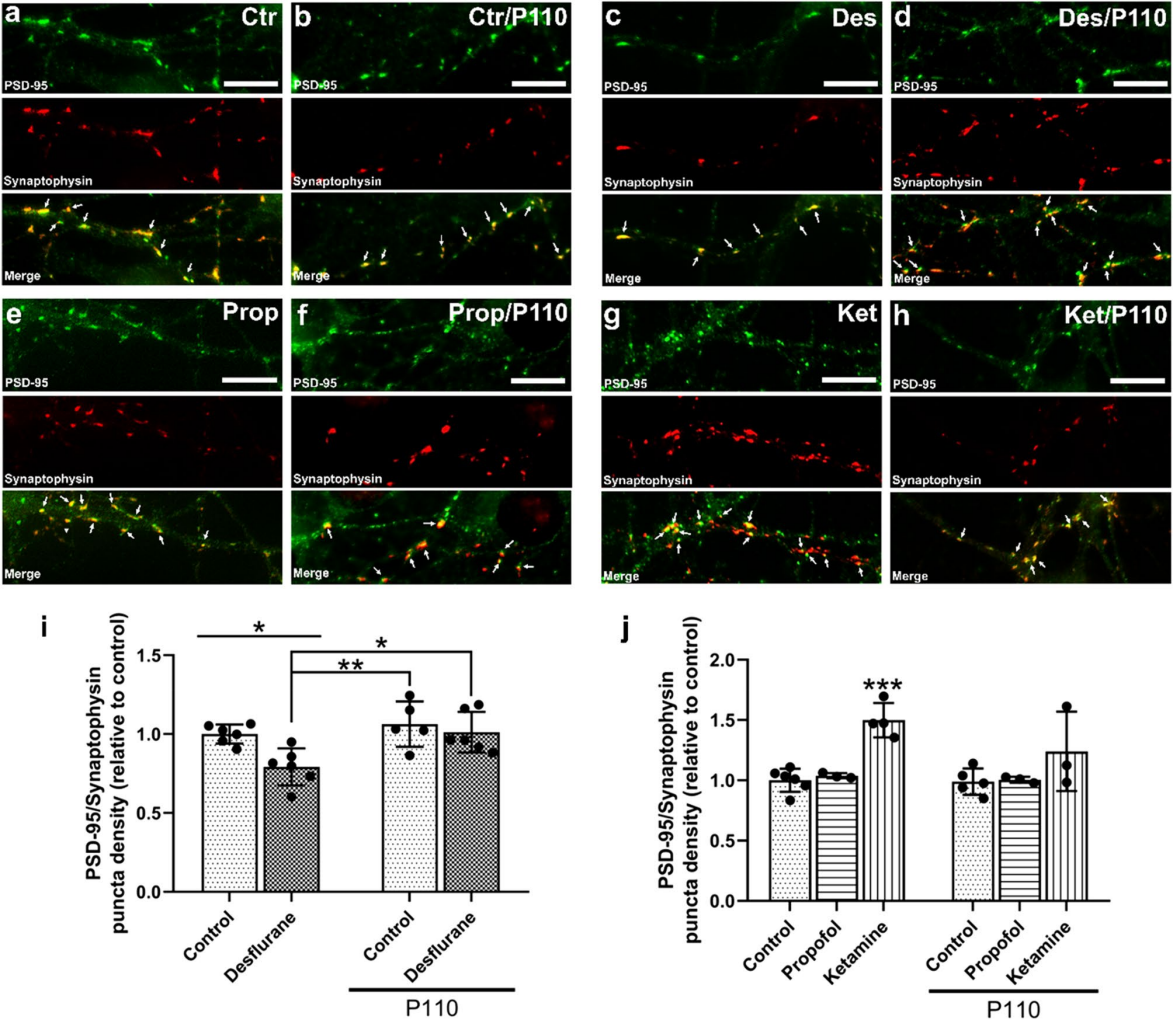
Desflurane but not propofol decreased synaptic protein expression whereas ketamine increased synaptic protein expression, P110 attenuated anesthetic-induced detrimental changes to synaptic assembly. Representative fluorescent images of cortical neurons stained for PSD-95 and synaptophysin at day in vitro 10 **(a–h)**. Each arrow on the merged images indicates examples of synaptic puncta along the neurite lengths as identified by the juxtaposed expression of pre-synaptic and post-synaptic labels that result in a distinctly yellow overlapping region. Quantification of average PSD-95/synaptophysin puncta per neurite length relative to control **(i–j)**. Exposure to 4.3 Vol% desflurane decreased average synaptic puncta density. Treatment with 10 µM propofol did not affect average synaptic puncta density. Treatment with 5 µM ketamine increased average puncta density. P110 alone did not affect synaptic network assembly, whereas its pre-treatment mitigated desflurane-induced pertubations and partially prevented ketamine-induced synaptogenesis. P110 followed by propofol treatment did not differ from control. Two-Way ANOVA. * *p* < 0.05, ** *p* < 0.01, *** *p* < 0.001, as determined by pairwise comparison of post-hoc tests. Bars indicate ± SD measured across biological replicates. Each data point indicates the mean synapse density per biological replicate. Scale bars indicate 5 μm.

Robust synaptic network assembly is prerequisite to proper brain development. To examine the effects of various anesthetics on synaptic network assembly, cells were stained for two synaptic proteins—synaptophysin, a presynaptic vesicle protein, and postsynaptic density protein (PSD-95); their expression patterns and colocalized juxtapositions were taken as an index of synaptic specialization (Figure S3). Only puncta that met these requirements were counted as synapses, such as the example highlighted in Figure S3. Instances where there was only synaptophysin signal (examples indicated with an arrow in Figure S3a), or only PSD-95 signal (examples indicated with an asterisk in Figure S3a) with no juxtaposition to each other and no overlapping yellow signal were not considered as the indicators of a synapse. Neurons exhibiting juxtaposition of PSD-95 (green) with synaptophysin puncta (red) were deemed as ‘synapses’ (Fig. 4a–h). Desflurane-exposed cells exhibited decreased density of synaptic puncta (*M* = 0.79, *SD* = 0.12 fraction of control, *p* = 0.0266, n = 6, *d* = 2.2136) compared to control (*M* = 1.00, *SD* = 0.06 fraction of control, n = 6) **(**Fig. 4i). Propofol, however, did not affect synaptic puncta density (*M* = 1.04, *SD* = 0.02 fraction of control, *p* = 0.9989, n = 3, *d* = 0.5547) compared to control (*M* = 1.00, *SD* = 0.1 fraction of control, n = 6) (Fig. 4j). Interestingly, ketamine-treated cells exhibited increased synaptic puncta density compared to control (*M* = 1.5, *SD* = 0.14 fraction of control, *p* = 0.0005, n = 4, *d* = 4.1100) and compared to those treated with propofol alone (*p* = 0.0062) (Fig. 4j). These data thus show that desflurane decreases synaptic puncta formation, whereas propofol does not significantly affect synaptic assembly. Contrary to the GABAergic desflurane, ketamine increased synaptic puncta density as revealed by the expression of two key synaptic markers, synaptophysin and PSD-95 in cortical neurons.

### Propofol and ketamine compromise mitochondrial networks whereas ketamine elevates mitochondrial superoxide production

**Figure 5:**
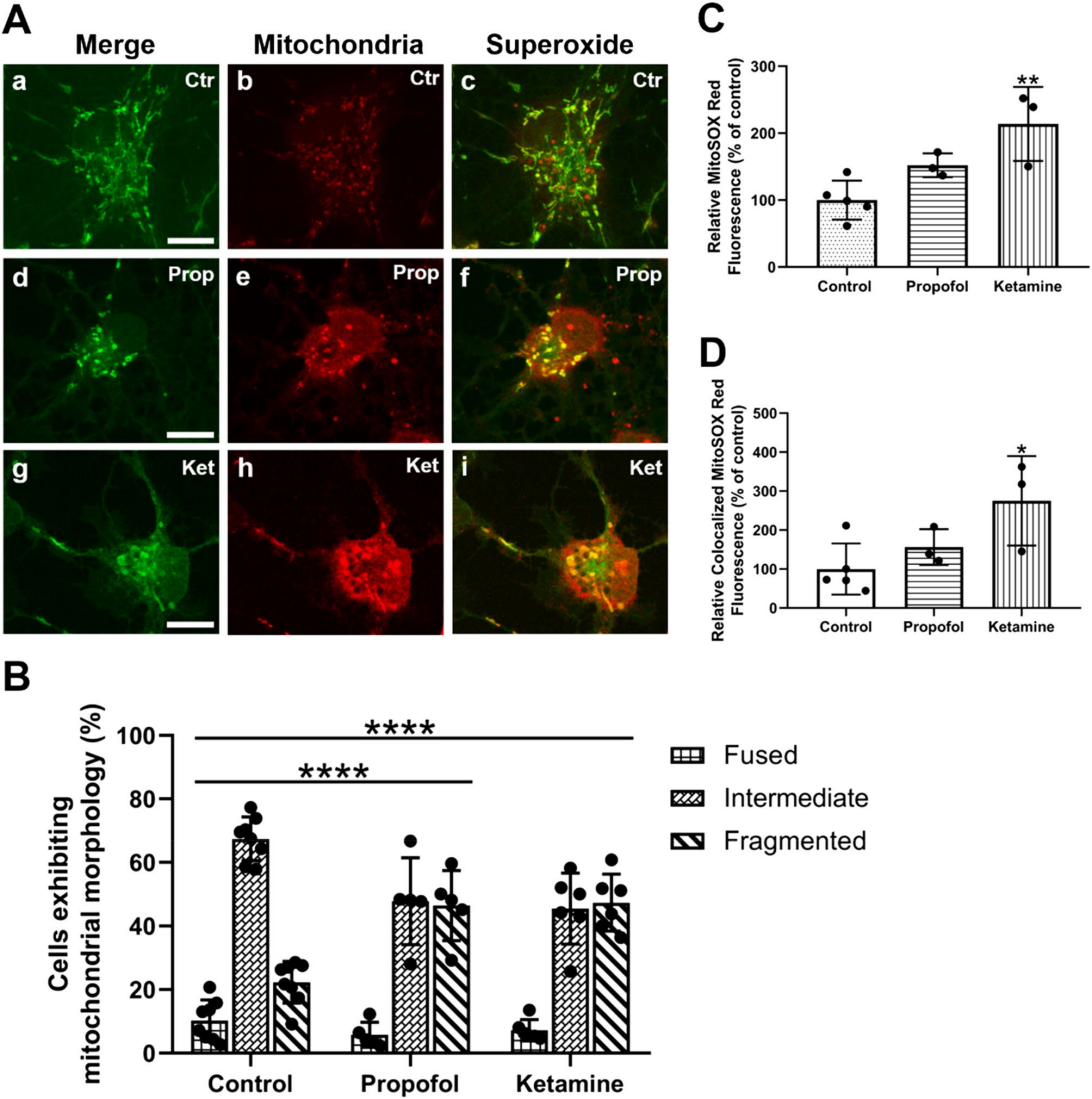
Propofol and ketamine induce mitochondrial fragmentation and ketamine dramatically increases SO production in primary cortical neurons. Representative cropped fluorescent images showing neurons from each condition which were co-stained with MitoTracker Green FM for mitochondria and the mitochondrial SO indicator MitoSOX Red **(A)**. Quantification of predominant mitochondrial morphology in each treatment. Both propofol and ketamine caused an increase in the proportion of cells exhibiting fragmented mitochondrial networks **(B)**. Quantification of relative SO production **(C)**. Propofol-treated neurons did not significantly differ from control, whereas ketamine more than doubled relative SO production. Quantification of relative SO based on MitoSOX Red signal which colocalized to MitoTracker Green FM **(D)**. Propofol did not significantly impact mitochondrial SO production, while ketamine almost tripled mitochondrial SO production compared to control. One-way or two-way ANOVA. * *p* < 0.05, ** *p* < 0.01, **** *p* < 0.0001 as determined by pairwise comparison of post-hoc tests. Bars indicate ± SD across at least biological replicates. Each data-point indicates the either the percentage of cells that exhibited a specific morphology per dish, or the mean SO signal recorded per dish. Scale bars indicate 10 μm.

All cellular processes depend on underlying energy dynamics, and thus the mitochondria have become the centre of extensive investigation as a potential target site for anesthetic-induced neurotoxicity and neurodegeneration. To assess the relative effects of the two intravenous agents propofol and ketamine on mitochondrial morphology, and to determine if any of the observed changes were accompanied by functional deficits, we examined SO production, which is an indicator of mitochondrial and cellular health. We labeled the neurons with MitoTracker Green FM for mitochondria and MitoSOX Red for SO production and discovered that under control conditions, mitochondria exhibited typical, elongated, rod-like morphology (Fig. 5A). Conversely, neurons exposed to anesthetics exhibited small, round morphology, with fewer elongated rod-like mitochondria, which is a characteristic of fragmented mitochondrial networks and mitochondrial toxicity, dysfunction, and the invocation of intrinsic apoptotic pathway (Fig. 5A). To validate these observations, we quantified the mitochondrial morphology in cortical neurons using a 3-point scale (fragmented, intermediate, and fused) (Fig. 1) and assessed the data via two-way ANOVA. We found that both propofol (*M* = 46.26%, *SD* = 11.07%, *p* < 0.0001, n = 5, *d* = 2.6281) and ketamine (*M* = 47.312%, *SD* = 8.98%, *p* < 0.0001, n = 6, *d* = 3.1719) caused aberrant mitochondrial fragmentation compared to control (*M* = 22.29%, *SD* = 6.62%, n = 8), with these conditions showcasing a higher proportion of cells with fragmented mitochondrial networks (Fig. 5B). We concomitantly examined SO production under these experimental conditions to quantify potential differences in the effects propofol and ketamine on mitochondrial function. By measuring fluorescence intensity of MitoSOX Red over the full field of view, we found that ketamine (*M* = 213.19%, *SD* = 55.35% of control, *p* = 0.0059 compared to control, n = 3, *d* = 2.5593), but not propofol (*M* = 152.1%, *SD* = 17.68% of control, *p* = 0.1736 compared to control, *p* = 0.146 compared to ketamine alone, n = 3, *d* = 2.1623) caused a dramatic increase in SO production in the cells compared to control (*M* = 100%, *SD* = 29.13% of control, n = 3) (Fig. 5C). We further investigated this relationship and quantified superoxide signal that colocalized with the mitochondrial signal. We found similar trends for both propofol (*M* = 156.3%, *SD* = 46.17% of control, *p* = 0.5981, n = 3, *d* = 0.99436) and ketamine (*M* = 275%, *SD* = 114.7% of control, *p* = 0.0348 compared to control and *p* = 0.2051 compared to propofol alone, n = 3, *d* = 1.8743) compared to control (*M* = 100%, *SD* = 65.42% of control, n = 3) (Fig. 5D). These data thus show both propofol and ketamine cause a robust increase in mitochondrial fragmentation and ketamine increases SO production.

### A synthetic peptide prevents desflurane-induced mitochondrial fragmentation

Previous studies have attempted to identify protective agents that may mitigate various aspects of anesthetic neurotoxicity. Specifically, we have previously demonstrated that desflurane-induced fragmentation is prevented by pre-treatment of cells with a mitochondrial fission inhibitor, mdivi-1^35^. However, the off-target effects of this drug range from inhibiting cellular proliferation to respiratory impairment and inhibition of basal levels of fission; these negative attributes thus negate its clinical potential and also prevent us from identifying the specific fission protein interactions that may be affected by anesthetics^25,46,47^. Instead, here we used a rationally designed synthetic peptide^45^ that targets excessive “pathological” mitochondrial fission by selectively blocking the dynamin-1-like protein (Drp1) and mitochondrial fission 1 protein (Fis1) interaction, which is upregulated during cellular stress. Blocking only this and not Drp1-receptor interactions that mediate physiological mitochondrial fission was deemed by us to help further elucidate the mechanisms of anesthetic neurotoxicity.

**Figure 6:**
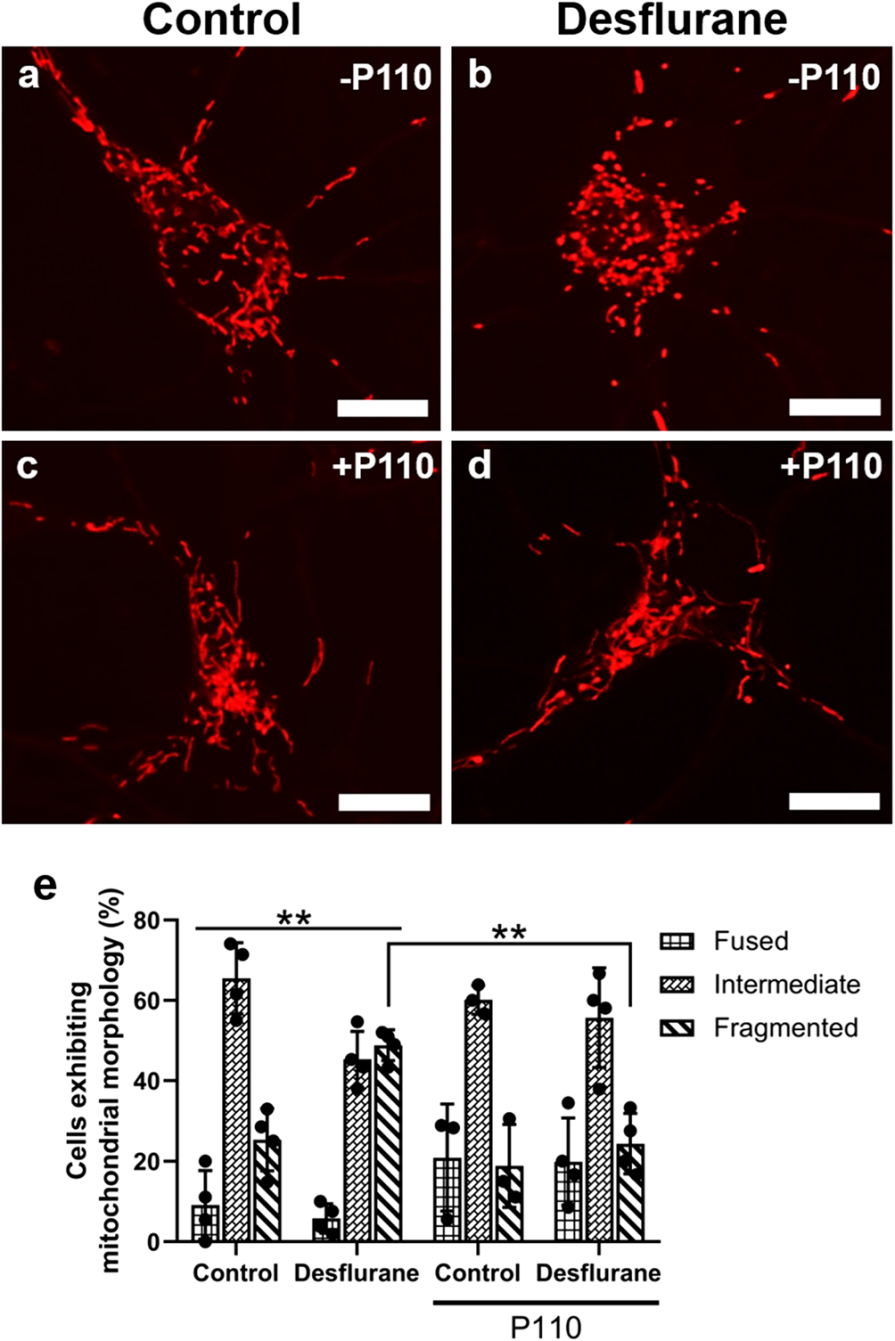
P110 treatment prevents desflurane-induced perturbation of mitochondrial morphology. Fluorescent cropped images showing representative neurons from each condition **(a–d)**. Quantification of predominant mitochondrial morphology in each treatment **(e)**. Desflurane alone caused increased fragmentation. P110 alone did not affect mitochondrial morphologies. P110 pre-treatment, followed by desflurane exposure prevented anesthetic-induced aberrations. Two-Way ANOVA. ** *p* < 0.001 as determined by Tukey's multiple comparisons post-hoc test. Bars indicate ± SD across biological replicates. Each data-point indicates the percentage of cells that exhibited a specific morphology per biological replicate. Scale bars indicate 10 μm.

We first investigated whether pre-treatment with the Drp1/Fis1 fission inhibitor P110 could help maintain mitochondrial network integrity both by itself and from desflurane-induced mitochondrial fragmentation. For this test, neurons were either treated with P110 (1 µM) mixed in culture media or media alone (control) on the day of cell plating and then exposed either to 4.3 Vol.% desflurane mixed with medical air, or medical air alone for 1 h. This concentration of P110 corresponds to lower-end dosage reported in literature^45^, and was validated further with tests in our lab (Figure S1). The cells were then maintained in culture and imaged after 4 days using Mitotracker Red CMXRos. Control neurons mostly exhibited typical, rod-like morphology between 0.75 and 3 µm in size (Fig. 6a). Desflurane-exposed cells, on the other hand, exhibited smaller, more rounded mitochondrial morphologies (Fig. 6b). Neurons treated with P110 appeared indistinguishable from control (Fig. 6c,d). To validate these observations further, we quantified the neurons based on mitochondrial morphologies using a 3-point fragmentation scale (fragmented, intermediate, and fused) (Fig. 1) and assessed the data via two-way ANOVA. These results were confirmed with post-hoc tests. We found that the cells exposed to clinical levels of desflurane exhibited increased fragmentation (control mean = 25.34%, *SD* = 7.74%, n = 4; desflurane alone mean = 48.85%, *SD* = 3.83%, *p* = 0.0026, n = 4, *d* = 3.8501). On the other hand, the morphological signatures of cells treated with P110 remained unchanged when compared to control (fragmented: *M* = 18.89%, *SD* = 10.29%, *p* = 0.7601, n = 3, *d* = 0.7084). Remarkably, cells pre-treated with P110 and then exposed to desflurane exhibited no significant difference from control or P110 alone (fragmented: *M* = 24.4%, *SD* = 7.51%, *p* = 0.9986 compared to control *d* = 0.1233, *p* = 0.8354 compared to P110 alone, n = 4, *d* = 0.6117). The morphological signatures did, however, differ from cells exposed to desflurane alone (*p* = 0.0017) (Fig. 6e). These data demonstrate that desflurane increased fragmentation of the mitochondria in primary rat cortical neurons, whereas P110 did not elicit any mitochondrial toxicity by itself. Furthermore, P110 pre-treatment attenuated desflurane-induced fragmentation, thus preserving mitochondrial networks.

### The synthetic peptide P110 reverses propofol-induced mitochondrial but not ketamine-induced fragmentation and superoxide production

**Figure 7:**
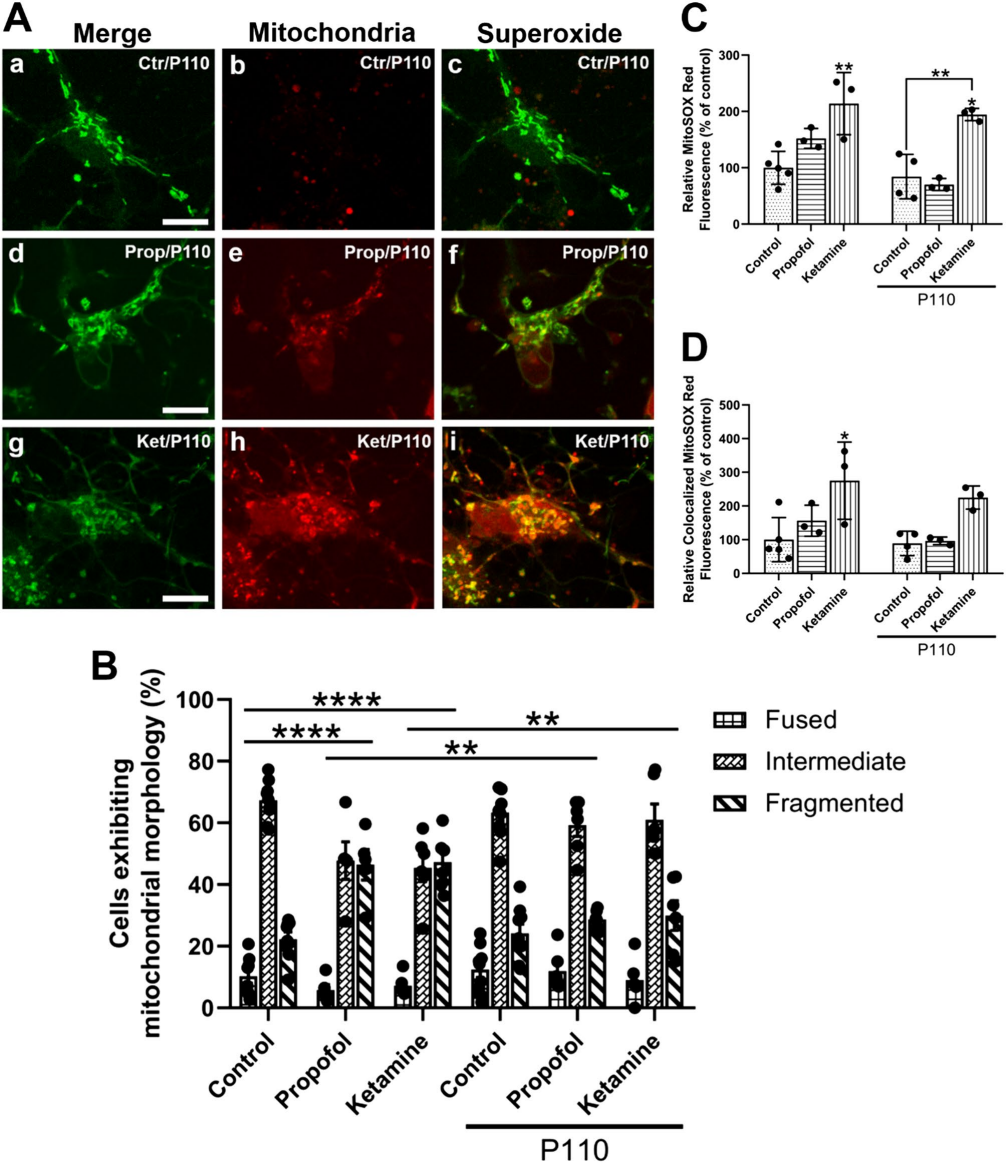
P110 reverses propofol-induced SO production and mitochondrial fragmentation. Representative cropped fluorescent images showing neurons from each condition which are co-stained with MitoTracker Green FM for mitochondria and the SO indicator MitoSOX Red **(A)**. Quantification of predominant mitochondrial morphology in each treatment **(B)**. For the ease of comparison, the control and anesthetic alone data are recapitulated here. P110 alone did not affect mitochondrial morphology whereas P110 pre-treatment fully rescued against propofol and ketamine-induced fragmentation. Quantification of relative SO production. For the ease of comparison, the control and anesthetic alone data are recapitulated here **(C)**. P110 alone did not affect SO production, however, P110 pre-treatment completely protected against propofol-induced SO production but not ketamine-induced SO production. Quantification of relative SO based on MitoSOX Red signal which colocalized to MitoTracker Green FM **(D)**. For the ease of comparison, the control and anesthetic alone data are recapitulated here. P110 reversed propofol-induced elevation in mitochondrial SO production but not ketamine-induced changes. Two-Way ANOVA. * *p* < 0.05, ** *p* < 0.01, **** *p* < 0.0001 as determined by pairwise comparison of post-hoc tests. Bars indicate ± SD across at least biological replicates. Each data-point indicates the either the percentage of cells that exhibited a specific morphology per dish, or the mean SO signal recorded per dish. Scale bars indicate 10 μm.

We further examined whether pre-treatment with the Drp1/Fis1 fission inhibitor P110 could help mitigate any changes in SO production and mitochondrial morphology caused by propofol and ketamine (Fig. 7A). We have observed above that both propofol and ketamine caused excessive mitochondrial fragmentation and that neurons treated with P110 alone did not show any differences from those of the control (fragmented: *M* = 24.19%, *SD* = 8.74%, *p* = 0.9972, n = 9, *d* = 0. 2451). Remarkably, P110 pre-treatment prevented excessive mitochondrial fragmentation caused by propofol and preserved the overall morphological signature as seen in control (*M* = 28.75%, *SD* = 3.04%, *p* = 0.7155 compared to control, *p* = 0.9081 compared to P110 alone, *p* = 0.0098 compared to propofol alone, n = 6, *d* = 1.2541) (Fig. 7B). Neurons treated with P110 and followed by ketamine treatment also appeared similar to control and were significantly less fragmented than those treated with ketamine alone (*M* = 29.95%, *SD* = 11.86%, *p* = 0.5455 compared to control, *p* = 0.7857 compared to P110 alone, *p* = 0.007 compared to ketamine alone, n = 6, *d* = 0.7976) (Fig. 7B). Taken together, these data demonstrate that pre-treatment with the Drp1/Fis1 fission inhibitor P110 protects primary cortical neurons from propofol-induced and ketamine-induced mitochondrial fragmentation.

Neurons treated with P110 alone displayed no change in SO levels as compared to control (*M* = 84.30%, *SD* = 39.46% of control, *p* > 0.9999 compared to control, n = 4, *d* = 0.42485). Cells treated with P110 followed by propofol exhibited SO expression which was not significantly different from either control, P110 alone, or propofol alone (*M* = 70.20%, *SD* = 10.69% of control, *p* = 0.9762 compared to control, *p* > 0.9999 compared to P110 alone, *p* = 0.0961 compared to propofol alone, n = 3, *d* = 5.6061) (Fig. 7C). P110 did not reverse ketamine-induced SO production (*M* = 194.48%, *SD* = 10.88% of control, *p* = 0.0155 compared to control, *p* = 0.0061 compared to P110 alone, *p* > 0.9999 compared to ketamine alone, n = 3, *d* = 0.46907) (Fig. 7C). Of these, the SO signal that colocalized with the mitochondrial signal showed similar trends. P110 by itself did not impact mitochondrial SO (*M* = 88.80%, *SD* = 36.29% of control, *p* = 0.9997, n = 4, *d* = 0.21172) compared to control (*M* = 100%, *SD* = 65.42% of control, n = 5). The peptide also did not impact propofol-induced SO production (*M* = 96.34%, *SD* = 11.24% of control, *p* > 0.9999 compared to control, *p* = 0.8202 compared to propofol alone, n = 3, *d* = 1.7845). P110 did not significantly impact ketamine-induced SO production, and was not significantly different from control either (*M* = 224.96%, *SD* = 34.43% of control, *p* = 0.1036 compared to control, *p* = 0.0839 compared to P110 alone, *p* = 0.9041 compared to ketamine alone, n = 3, *d* = 0.5909) (Fig. 7D). Together, these data demonstrate that the synthetic Drp/Fis1 fission inhibitor P110 did not affect mitochondrial SO production by itself and prevented propofol but not ketamine-induced changes to mitochondrial morphology when administered concomitantly with the tested anesthetic (Fig. 7).

### P110 attenuates anesthetic-induced cell death in primary cortical cultures

To validate further the neuroprotective properties of P110 against anesthetic-induced cell death, we next assessed cell viability of the neuronal cultures. Two-way ANOVA and subsequent post-hoc tests showed that cells treated with P110 alone maintained survival rates similar to those of control (*M* = 85.12%, *SD* = 2.5% alive, *p* = 0.9622 compared to control, n = 6, *d* = 0.4666). Remarkably, cells pre-treated with P110 followed by desflurane exposure exhibited no change from control or P110 alone in terms of viable cells and were significantly better than those exposed to desflurane alone (*M* = 82.61%, *SD* = 2.74% alive, *p* = 0.9958 compared to control, *p* = 0.7169 compared to P110 alone, *p* = 0.0206 compared to desflurane alone, n = 6, *d* = 0.2953) (Fig. 2j). These data demonstrate that exposure to 4.3 Vol.% desflurane results in significant neuronal death in vitro, whereas P110 pre-treatment provides neuroprotection against desflurane-induced death of primary rat cortical neurons.

Similar observations were made for intravenous agents, wherein cells treated with P110 alone maintained cell viability (*M* = 76.85%, *SD* = 8.1%, *p* = 0.846 compared to control, n = 10, *d* = 0.5723). Neurons treated with P110 followed by propofol exposure exhibited no change from that of the control or P110 alone, although it was not significantly higher than cells treated with propofol alone either (*M* = 69.29%, *SD* = 9.85% alive, *p* = 0.1242 compared to control, *p* = 0.6255 compared to P110 alone, *p* = 0.5395 compared to propofol alone, n = 5 areas, *d* = 1.3975) (Fig. 2l). P110 followed by ketamine exposure did not differ from controls either (*M* = 79.63%, *SD* = 7.6% alive, *p* = 0.9993 compared to control, *p* = 0.9596 compared to P110 alone, *p* = 0.9291 compared to ketamine alone, n = 6, *d* = 0.2089) (Fig. 2l). These data demonstrate that P110 may provide protection against the neurotoxic effects propofol in vitro*.*

### P110 rescues desflurane, propofol, and ketamine-induced effects on neurite outgrowth and synaptogenesis

#### P110 rescues anesthetic-induced changes in cortical neurite outgrowth

To validate the neuroprotective potential of P110, we next tested the effects of desflurane, propofol, and ketamine on the extent of growth after pre-treatment with P110. Cells treated with P110 alone (*M* = 0.96, *SD* = 0.04 fraction of control, *p* = 0.9794 compared to control, n = 3, *d* = 0.34300) and those treated with P110 followed by desflurane exposure showed no significant change from control (*M* = 1.01, *SD* = 0.16 fraction of control, *p* = 0.9992 compared to control, *p* = 0.9536 compred to P110 alone, *p* = 0.0553 compared to desflurane alone, n = 3, *d* = 2.3882) (Fig. 3). Similarly, we discovered that propofol did not affect neurite outgrowth in developing primary cortical neurons. Neurons treated with P110 alone (*M* = 0.90, *SD* = 0.09 fraction of control, *p* = 0.9908 compared to control, n = 3, *d* = 0.8944), or P110 followed by propofol treatment also showed no deviation from control (*M* = 0.95, *SD* = 0.09 fraction of control, *p* > 0.9999 compared to control, *p* > 0.9999 compared to P110 alone, *p* = 0.9962 compared to propofol alone, n = 3, *d* = 1.7857) (Fig. 3).

Neurons treated with ketamine exhibited extensive growth compared to control, whereas pre-treatment with P110 attenuated the excessive growth seen in ketamine-treated cells and showed no difference from control (*M* = 1.11, *SD* = 0.02 fraction of control, *p* = 0.9975 compared to control, *p* = 0.6732 compared to P110 alone, *p* < 0.0001 compared to ketamine alone, n = 3, *d* = 3.1379) (Fig. 3). Taken together, these data show that ketamine enhances neurite outgrowth of developing cortical neurons, and that these effects are also reversed by P110 pre-treatment.

### P110 attenuates anesthetic-induced changes in synaptic network assembly in cortical neurons

We next asked whether P110 could provide neuroprotection against anesthetic-induced toxicity regarding its ability to preserve neuronal network health and synaptic assembly. We found that P110 treatment did not affect total synaptic puncta density by itself (*M* = 1.06, *SD* = 0.14 fraction of control, *p* = 0.8034 compared to control, n = 5, *d* = 0.5571), rather it reversed the desflurane-induced decrease in density of synaptic puncta (*M* = 1.01, *SD* = 0.13 fraction of control, *p* = 0.9979 compared to control, *p* = 0.8828 compared to P110 alone, *p* = 0.0182 compared to desflurane alone, n = 6, *d* = 0.09877) (Fig. 4). Accordingly, P110 treatment alone (*M* = 0.99, *SD* = 0.11 fraction of control, *p* > 0.9999 compared to control, n = 5, *d* = 0.09513) or followed by propofol treatment did not change synaptic puncta density compared to control in the intravenous anesthetic tests (*M* = 1.00, *SD* = 0.03 fraction of control, *p* > 0.9999 compared to control, *p* > 0.9999 compared to P110 alone, *p* = 0.9997 compared to propofol alone, n = 3, *d* = 1.5689) (Fig. 4). Interestingly, P110 pre-treatment followed by ketamine exposure did not differ from control cells, or to the synonymous P110 alone cells (*M* = 1.24, *SD* = 0.33 fraction of control, *p* = 0.2237 compared to control, *p* = 0.2114 compared to P110 alone, *p* = 0.2244 compared to ketamine alone, n = 3, *d* = 1.0257) (Fig. 4). Taken together, these data show for the first time, that treatment with the mitochondrial fission inhibitor peptide P110 allows us to rescue neurons from anesthetic-mediated reduction in synaptic puncta density in cortical neurons while completely reversing desflurane-induced decreases in the number of boutons, and partially preventing ketamine-induced increases.

## Discussion

There is now significant awareness of the potential neurotoxic effects of anesthetic agents on brain development, and progress has been made in understanding the mechanisms underlying the evidence of harm^13,21,22^. However, despite several studies investigating these effects, developing a consensus vis-à-vis the effects of anesthetics on neuronal health has remained elusive. The reasons for this lack of consensus perhaps owe their existence to the fact that myriad models have been subjected to different anesthetic agents and under diverse experimental conditions, thus generating irreconcilable conclusions. As such, neither the precise mechanisms, nor the extent to which these effects may impact any particular patient can be considered reliably. Hence, we deemed it important to resolve these differing conclusions by creating identical experimental conditions through which to test the effects of key inhalational and intravenous general anesthetics on developing neural circuits. We selected this experimental paradigm for its versatility and specificity. The primary culture setup offered a higher resolution assessment of fundamental characteristics ranging from the initiation of growth to forming of synaptic networks during critical “neurodevelopmental” periods which could not otherwise be monitored in vivo. Specifically, such an analysis at the cellular and molecular level is not feasible in intact, freely behaving animals.

Interestingly, we discovered that under identical cortical cell culture conditions, desflurane, propofol, and ketamine exerted differing effects on neurite growth and synaptic network assembly. While the GABAergic anesthetic agent desflurane decreased synaptic density, the NMDAergic ketamine had the opposite effect, whereas the GABAergic propofol had no significant impact on these parameters. This suggests an agent-specific influence on key characteristics of neuronal development. Even with these differing effects however, both desflurane and propofol caused significant cell death. These observations are consistent with previous in vitro studies using either the same or lower concentrations^35,53,54^. We do not however rule out the possibility that dead fibroblasts or glia may have also been included in our assessment—notwithstanding concerted efforts to count only those cells with larger nuclei and morphological features that are characterises of neurons. Moreover, we monitored here only fresh cultures where the glial count is the lowest (due to their smaller sizes, they take much longer time to adhere to the substrate than neurons). A potential caveat however is that at such an early stage of culture, neurons were exposed to drugs as opposed to later stages when networks of neurons would have been fully established and cells become less susceptible to membranous damage. While we showed morphological, and not functional attributes of a synapse, our previous experiments—conducted under identical conditions—have nevertheless demonstrated that decreased synaptic puncta are always corroborated with lower amplitude and frequency of spontaneous synaptic events^35^. The differences observed between agents thus support and build on previous studies that have investigated these drugs individually and substantiate the notion that regardless of the model system used, both inhalational and intravenous general anesthetics exert different effects on neuronal viability and growth.

For example, previous studies have demonstrated propofol-induced acute neurite retraction in primary rat cortical neurons (minutes after anesthetic exposure) which was completely reversed within 10 min after the washout for lower concentrations (0.02, 0.2, and 2 µM)^20^. Although these live-imaging tests are consistent with our observations, the authors noted that the effects of longer exposure times or exposure to 20 µM propofol could not be reversed. This may be explained by the short timeline of the experiment, with neurite lengths being observed within hours of anesthetic exposure, and not days afterwards to quantify lasting effects. The observation that ketamine increased neurite outgrowth at the above concentration was indeed surprising, but these data corroborate well with previous studies which have shown that this anesthetic, as well as some antidepressants, increased neurite outgrowth and dendritic spine density in hippocampal and cortical neurons, respectively^51,55^. This may be a result of an upregulation of mTOR signaling or other un-identified mechanisms. For the impact of propofol on synaptogenesis, divergent results have been reported with regards to cell type and the age of animals^12,56^. Similarly, ketamine has been previously shown to either downregulate synaptic proteins in the hippocampus of the developing brain^57^, or to upregulate them in the cortex^58^. A 2010 study demonstrated that activation of the mTOR pathway underlies ketamine-induced synaptogenesis by illustrating increased dendritic spine density in cortical neurons. This was coupled with a functional assessment of synaptic activity in pyramidal cells^55^. Likewise, the increase in neurite outgrowth and synaptic puncta density observed above seem to follow suit of reported effects of ketamine on glutamatergic transmission long after drug washout. Increased glutamate signaling post-anesthetic via the α-amino-3-hydroxy-5-methyl-4-isoxazole propionic acid (AMPA) receptor may explain part of its effects on increased number of synaptic structures that were discerned in the present study^59^. Further,

The widely documented GABA depolarizing-to-hyperpolarizing polarity switch in the developing brain^60,61^ may underlie some of the differences in the effects of these agents. GABAergic transmission plays an important role in propagating giant depolarizing potentials, which are crucial for early brain development and for additional GABAergic synaptogenesis^60,62^. We speculate that anesthetic-induced GABA agonism by agents such as isoflurane^63^, desflurane^64^, propofol^65,66^, and to a minor degree, ketamine^67^, may be a factor responsible for increasing GABAergic sensitivity in developing brains when administered to pregnant mother or neonates. In the context of apoptosis, this may underlie anesthetic-induced GABAergic excitotoxicity^63^ and the subsequent cell death. Acting in opposition to this, ketamine-mediated NMDA antagonism^68^ may counteract these effects, and provide protection against excitotoxicity^69^. Whether such protection may also be available to the developed brain where GABAergic currents become inhibitory, remains to be determined. Thus, various reports suggesting ketamine’s neuroprotective role in brains exposed to noxious stimuli cannot be discounted when investigating anesthetic neurotoxicity^70^. For instance, a 2018 study found that ketamine prevented behavioral deficits caused by isoflurane^71^, an agent that induces GABAergic excitotoxicity in the developing brain^63^. Preventing hyperactive synaptic transmission which may initiate cell death, and modulating mitochondrial function may complement ketamine’s mTOR-mediated anti-depressant properties^55^ in what would have otherwise been a highly excitotoxic cellular environment.

This dual function may also explain ketamine’s purported neuroprotective effects in the developing brain. Various investigators have commented on the seemingly opposite effects of ketamine and have correctly suggested that drug dose, frequency of exposure, and the noxious state of the brain may impact ketamine-induced neurotoxicity verses neuroprotection^72^. We believe that this mechanistic understanding may provide a clearer answer to these exciting set of questions, many of which remain elusive. For example, determining whether ketamine could by itself, relieve anesthetic-induced excitotoxicity may help explain its clinical uses. These insights could offer a unique opportunity to develop novel strategies to mitigate anesthetic-induced neurotoxicity in the clinic.

To identify a potential common mechanism underlying anesthetic-induced neurotoxicity in developing neurons, we investigated neuronal energy dynamics; specifically, we examined mitochondrial structure and function. Mitochondrial dysfunction has been previously shown to appear quickly (within two hours) following anesthetic exposure and leads to neuron apoptosis^3^. Furthermore, Boscolo, et al.^73^ showed that anesthetic-induced mitochondrial dysfunction in P7 rat pups also produced more reactive oxygen species (ROS), disintegrated inner mitochondrial membranes and resulted in cognitive dysfunction. These effects were, however, reversible when mitochondrial integrity was protected with R( +) pramipexole (PPX). R( +) PPX blocks the disintegration of the mitochondrial membrane by downregulating ROS^74,75^, thereby preventing activation of apoptotic pathways driven by excessive ROS^76^. While well-tolerated in high doses and relatively free of non-target effects^73^, R( +) PPX does not directly target underlying mitochondrial dysfunction. For these reasons, we opted to use a synthetic peptide, P110^45^, to selectively impair Drp1-mediated mitochondrial fission.

In recent years, the roles of disrupted mitochondrial networks and its compromised dynamics have also come to light in several neurodegenerative diseases, although clinical treatments remain limited^77^. Changes to cellular growth machinery and cytoskeletal protein function heavily involve these mitochondrial networks, and the delivery of functional mitochondria to sites with high metabolic requirements (such as growth cones and branch points) is crucial in synapse formation and synaptic transmission^78–80^. Thus, any perturbation of mitochondrial dynamics may have lasting implications on neuronal network development^81^. We reason that this may explain the trends observed in our results and the discrepancy in the field with regards to making a direct comparison between primarily NMDAergic ketamine and the GABAergic propofol. That said, questions remain as to how exactly ketamine influences mitochondrial integrity and superoxide production. Reconciling whether these may be through downstream effects of glutamate modulation, or NMDA receptor-based calcium influx, amongst other postulates provide an exciting avenue for further research^82,83^.

We have previously demonstrated that both desflurane and sevoflurane-induced neurotoxicity could be reversed by the fission inhibitor mdivi-1 using the same experimental paradigm^35^. These observations were consistent with an earlier study where propofol-induced cytotoxic effects on a cell line were found to be attenuated by mdivi-1^15^. However, the clinical applications of mdivi-1 are limited due to its off-target effects^84^. Our approach here was to selectively block Drp1/Fis1 interaction without interfering with the interactions of this cascade with other physiological functions. We showed here that using P110, a non-toxic, seven amino acid-long selective inhibitor of Drp1/Fis1^45^ not only attenuates anesthetic-induced neurotoxicity, but that it also rescues cultured cortical cells from desflurane, propofol, and ketamine-induced mitochondrial fragmentation. These results are the first to demonstrate the protective effects of P110 against anesthetic-induced neurotoxicity. To our knowledge this is also the first study to link Fis1 receptor-mediated fission with anesthetic-induced aberrations. Since the peptide protects against both GABAergic and NMDAergic agents (although there is some overlap in action^21^), our data suggest a common site of anesthetic actions targeting mitochondria. Regarding the potential mechanisms, it may be reasonable to suggest that changes in cellular energetics, hyperactivity, or other yet-to-be explored interactions of anesthetics with mitochondria might be involved. The data presented in this study has identified mitochondria as potential therapeutic target against anesthetic-induced cytotoxicity, but further research is required to identify how key mitochondrial cycles are regulated by anesthetics. Moreover, additional studies would need to be conducted to demonstrate that the anesthetic-induced effects on animal learning, memory and cognition could also be reversed by P110 peptide in freely behaving animals.

Taken together, anesthetic-induced neurotoxicity is a significant issue. However, given that the anesthetics are essential tools that enable critical surgeries, they are unavoidable. This does not however, mean that their cytotoxic and long-term effects should be overlooked as it would undermine our efforts directed at searching for least toxic anesthetic agents or to synthesize compounds that would mitigate their cytotoxic effects in humans. This study makes two important contributions to our understanding of anesthetics and neurotoxicity. First, we provide a pragmatic account of both commonly used inhalational and intravenous general anesthetic agents with different modes of action in relation to their effects on key aspects of neuronal health under identical experimental conditions. We propose a mechanistic-based paradigm to the varying results observed in the field. We integrate these findings with current interpretations of anesthetic-induced neurotoxicity and attempt to fill several gaps in our understanding of anesthetic-induced harm in cultured rat cortical neurons. Second, we identify a potential, novel protective strategy against anesthetic-induced neurotoxicity by preserving mitochondrial health. This approach could help design future neuroprotective drugs with potential clinical applications. We showed here that the neurotoxic effects of the anesthetics are either mitigated or reversed by a selective mitochondrial fission inhibitor, P110, showcasing both in vivo and clinical potential. The findings presented here thus underscore the fact that preserving mitochondrial networks may be a key for future research into potential neuroprotective agents that will complement the vital role of anesthetics in the operating theatre.

## Supplementary Information

Supplementary Information

## Data Availability

The data that support the findings of this study are openly available in the Mendeley Data Depository at 10.17632/5j76t2b374.1.
