# Supplementary material for: A synthetic peptide rescues rat cortical neurons from anesthetic-induced cell death, perturbation of growth and synaptic assembly
Source: Sci Rep. 2021 Feb 25;11:4567. doi: 10.1038/s41598-021-84168-y (PMC7907385; doi:10.1038/s41598-021-84168-y)
Supplement: Supplementary file 1 — Supplementary Information [file 41598_2021_84168_MOESM1_ESM.pdf]

Supplementary Material

A synthetic peptide rescues rat cortical neurons from anesthetic-induced cell death, perturbation of growth and synaptic assembly

Fahad Iqbal[1], Marcus Pehar[1†], Andrew J. Thompson[1†], Urva Azeem[1†], Kiana Jahanbakhsh[1†], Nerea Jimenez-Tellez[1], Rasha Sabouny[1], Shadab Batool[1], Atika Syeda[2], Jennifer Chow[1], Pranav Machiraju[1], Timothy Shutt[1], Kamran Yusuf[1], Jane Shearer[1], Tiffany Rice[1], Naweed I. Syed[1]*

[1]University of Calgary, [2]University of Oxford, [†] indicates equal contribution, * indicates corresponding author

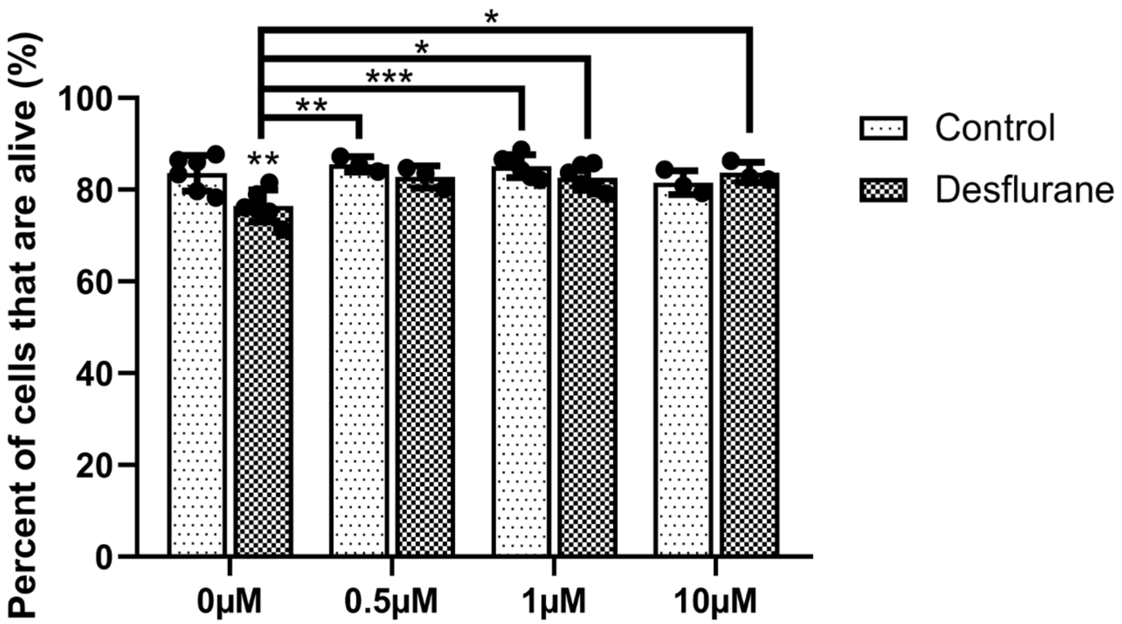

Figure S1: P110 prevents desflurane-induced cell death in a concentration-dependent manner. P110 did not affect cell viability by itself (0.5µM: $M = 85.52\%$, $SD = 1.66\%$ alive, $p > 0.9999$ compared to control, n = 3; 1µM: $M = 85.12\%$, $SD = 2.5\%$ alive, $p > 0.9999$ compared to control, n = 6; 10µM: $M = 81.52\%$, $SD = 2.59\%$ alive, $p > 0.9999$ compared to control, n = 3) and instead reversed desflurane-induced cell death (0.5µM: $M = 82.76\%$, $SD = 2.39\%$ alive, $p > 0.9999$ compared to control, $p = 0.1424$ compared to desflurane alone, n = 3; 1µM: $M = 82.61\%$, $SD = 2.74\%$ alive, $p > 0.9999$ compared to control, $p = 0.0347$ compared to desflurane alone, n = 6; 10µM: $M = 83.72\%$, $SD = 2.21\%$ alive, $p > 0.9999$ compared to control, $p = 0.0482$ compared to desflurane alone, n = 3). Two-Way ANOVA. * $p < 0.05$, ** $p < 0.01$, *** $p < 0.001$. Experiments and results were validated at least twice. Bars indicate ±SD across biological replicates. Each data-point indicates the mean viability reported in each biological replicate.

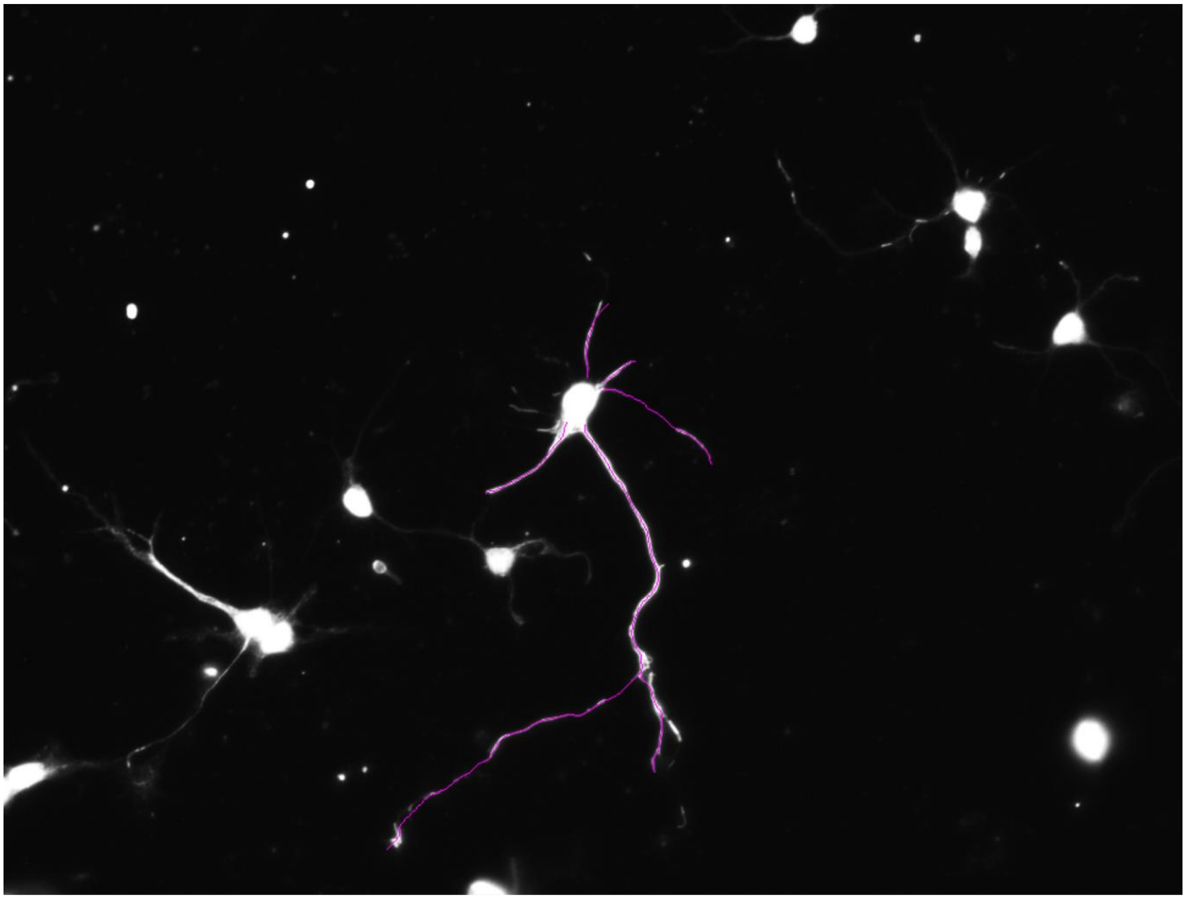

Figure S2: Example of neuronJ tracing of neurites of a cell at day *in vitro* 3. The central cell in each field of view following a standardized imaging protocol was traced and the average neurite lengths per aggregate for each biological replicate.

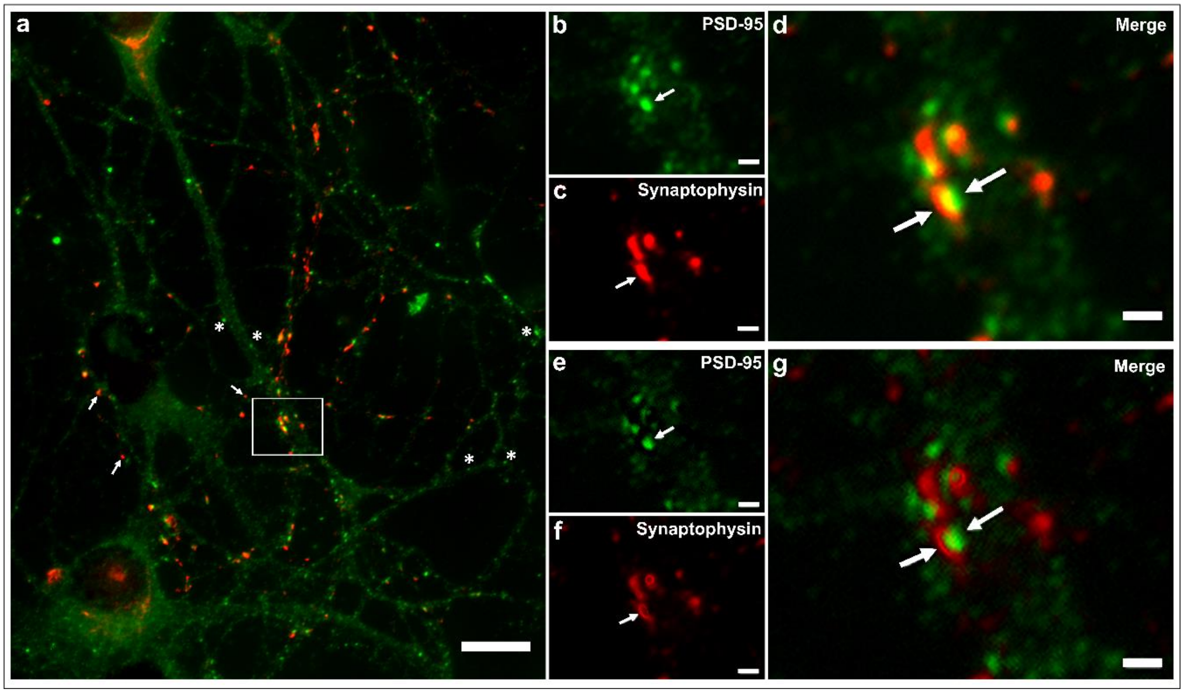

Figure S3: Example of synaptic network staining for quantification of synaptic density. Representative fluorescent image that is labelled for the post-synaptic marker PSD-95 (green) and the presynaptic marker synaptophysin (red) **(a)**. Close-up of a synaptic punctum where the juxtaposition of PSD-95 **(b)** and synaptophysin **(c)** produces a characteristic yellow, which was quantified as a punctum **(d)**. A sharpened image is shown in the final row to verify and display the localization of these signals at the synaptic site **(e-g)**. Average puncta density was manually quantified as number of puncta per unit length wherever these characters for a synapse were met. Scale bars indicate 10µm **(a)** and 1µm **(b-g)**.
